# Exogenous gibberellin can effectively and rapidly break intermediate physiological dormancy of *Amsonia elliptica* seeds

**DOI:** 10.3389/fpls.2022.1043897

**Published:** 2022-10-26

**Authors:** Sang Yeob Lee, Kyungtae Park, Bo-Kook Jang, Boran Ji, Hamin Lee, Carol C. Baskin, Ju-Sung Cho

**Affiliations:** ^1^ Kiban Operation Department (KOD) production planning, The Kiban Co. Ltd., Anseong, South Korea; ^2^ Division of Animal, Horticultural and Food Sciences, Chungbuk National University, Cheongju, South Korea; ^3^ Brain Korea 21 Center for Bio-Health Industry, Chungbuk National University, Cheongju, South Korea; ^4^ Garden and Plant Resources Division, Korea National Arboretum, Pocheon, South Korea; ^5^ Department of Biology, University of Kentucky, Lexington, KY, United States; ^6^ Department of Plant and Soil Sciences, University of Kentucky, Lexington, KY, United States

**Keywords:** Amsonia elliptica, physiological dormancy, seed scarification, dormancy break, moist stratification

## Abstract

Accelerated global warming is leading to the loss of plant species diversity, and *ex situ* preservation of seeds is becoming an increasingly important aspect of species conservation. However, information on dormancy and germination is lacking in many endangered species. *Amsonia elliptica* (Apocynaceae) is the only *Amsonia* species native to Korea, and the South Korean Ministry of Environment has designated it Class II endangered wildlife. Nevertheless, the dormancy class and the dormancy breaking method for seeds of this species for germination are not precisely known. We identified the structure of *A. elliptica* seeds and the causes of dormancy, which inhibits germination. In addition, we tried to develop an effective germination promotion method by testing the wet stratified condition, which breaks dormancy, and the form of gibberellin that can replace it. Fresh seeds of *A. elliptica* imbibe water, but the covering layers (endosperm and seed coat) inhibit germination by mechanically restricting the embryo. Initial germination tests confirmed low embryo growth potential and physiological dormancy (PD). Restriction due to the covering layer was eliminated by seed scarification, and abnormal germination was observed. After 12 weeks of cold moist stratification at 4°C, only 12% of seeds germinated. However, 68.8% of seeds subjected to 8 weeks of warm moist stratification followed by 12 weeks of cold stratification germinated, indicating that warm stratification pretreatment before cold stratification is effective in breaking dormancy. *A. elliptica* seeds exhibited intermediate PD. Furthermore, 61.3% of seeds soaked in 500 mg/L GA_4+7_ for 14 days and incubated at 25/15°C germinated. Therefore, GA_4+7_ rapidly broke the dormancy of *A. elliptica* seeds compared with warm plus cold stratification treatment, thus providing an efficient method for seedling production.

## Introduction

Ecosystems are rapidly changing owing to various environmental problems and global warming, and one of the most important issues is the loss of plant species diversity (Walther et al., 2002). Various *in situ* and *ex situ* efforts have been made worldwide to conserve plant diversity (Hawkins et al., 2008; Li and Pritchard, 2009). A common *ex situ* conservation strategy for plant species involves storing seeds in seed banks, which allows several genetic resources to be protected in a relatively small space (Cochrane et al., 2007; Kramer and Havens, 2009; Hay and Probert, 2013). However, collecting and storing seeds does not necessarily solve the conservation issue because the seeds must eventually germinate if they are to be used for restoration. For many species, however, we do not have information about their dormancy-breaking and germination requirements, which are essential for plant propagation from seeds. Therefore, understanding the dormancy and germination characteristics of seeds is as important for restoration as collecting and storing a diverse and large number of species.

Seed dormancy means germination does not occur even if environmental conditions are suitable for germinating non-dormant seeds (Hilhorst, 1995; Baskin and Baskin, 2014). Dormancy may be due to characteristics (blocks) of the embryo (e.g., underdevelopment and/or a physiological inhibiting mechanism) and/or the seed/fruit coat, and these blocks must be removed or overcome before germination can occur (Baskin and Baskin, 2014). To break dormancy, seeds must be exposed to certain environmental conditions, which vary depending on the dormancy type (class). There are five classes of dormancy, which may be subdivided into levels, e.g., physiological dormancy (PD) is divided into three levels (Nikolaeva, 2004; Baskin and Baskin, 2021). Therefore, knowledge of the dormancy classes and how they can be overcome is useful for plant propagation and utilization and is especially essential for species conservation.

*Amsonia* is a genus in the Apocynaceae family that is widely distributed from the southern United States to northwestern Mexico and eastern Asia. *Amsonia elliptica* is the only *Amsonia* species in Korea, and it is a plant with great ornamental and medicinal potential (Sauerwein et al., 1991; Kim et al., 2006; Yang et al., 2012). However, *A. elliptica* has been designated Class II endangered wildlife by the South Korean Ministry of Environment because of its rarity in nature and the loss and destruction of its habitat. Therefore, information on the seed dormancy break and germination requirements of this species is important for its conservation and restoration.

Germination has been reported to improve when seeds of *Amsonia tabernaemontana* are scarified and then sown (Martin et al., 2013). In addition, according to a patent published in Korea (No. 10-2017-0078854), the germination of *A. elliptica* is improved by chemical scarification (Choi and Jung, 2017). Thus, based on the available information, we can conclude that the seed coat of *Amsonia* species inhibits germination. Seed coat inhibition of germination could be due to the water impermeability of the seed coat, i.e., physical dormancy (PY), or to the low growth potential of the embryo, i.e., PD.

The hormone gibberellin (GA) is a major regulator of seed germination and is controlled by various factors within the seed (Vishal and Kumar, 2018). Before the germination process begins, abscisic acid levels are downregulated, whilst the GA content is upregulated (Shu et al., 2016; Urbanova and Leubner-Metzger, 2016). This increase in GA occurs with the release of seed dormancy, such as imbibition and stratification. Treatment with exogenous GA in some dormant seeds can use this opportunity to break dormancy, allowing for faster seedling production. Exogenous GA treatment was effective for breaking dormancy in *Cinnamomum migao* (Chen et al., 2022), *Leymus chinensis* (He et al., 2016), and *Thalictrum Uchiyama* (Lee et al., 2018) seeds, although *Clematis terniflora* (Jang et al., 2023) and *Ferrula assa-foetida* (Hassani et al., 2009) seeds were unaffected. Many previous studies have applied GA to promote dormancy breakage, although responses to this have shown variation with plant species, dormancy class, forms of GA, concentration, and treatment length.

However, to the best of our knowledge, currently no information is available regarding the exact dormancy class of *A. elliptica*. Therefore, our study aimed to classify dormancy in *A. elliptica* seeds and furthermore, to identify the conditions required to break it. Specifically, we determined whether the seeds were water-permeable and evaluated their responses to scarification, cold and warm (moist) stratification, and treatment with GA. Our results provide valuable information for the stable preservation and restoration of seeds and could potentially be used to prevent the extinction of *A. elliptica*, through stable seedling production and distribution.

## Materials and methods

### Plant material

Ripe seeds were collected in November 2019 from *A. elliptica* plants growing in Miwon-myun, Cheongju-si, Chungcheongbuk-do, Korea. The seeds were cleaned by hand, sieved to remove plant debris, and stored at 4°C until use in the experiments.

### Seed properties and initial germination test

An imbibition test was performed immediately after collection to determine whether the *A. elliptica* seeds could imbibe water. First, after determining the weight of 4 replicates of 50 seeds each, the seeds were placed in 15-mL tubes with distilled water. After immersion for 24 h, the seeds were removed from the water, dried with a towel, weighed, and immersed in distilled water again. The same procedure was repeated for 7 days. The increase in mass was calculated based on the initial weight. Scarified seeds were also used to double-check for impermeability of the covering layer.

The viability of fresh seeds was tested using 1% tetrazolium (triphenyl tetrazolium chloride, TTC; Sigma-Aldrich, St Louis, MO, USA) and X-ray scanning. The tetrazolium test was performed on 100 seeds soaked in distilled water at 20°C for 2 days. After soaking in water, the seeds were cut in half and placed on filter paper (Whatman no.1, Cytiva, Marlborough, MA, USA) moistened with tetrazolium solution in a 100-mm Petri dish cut-side-down for 1 day. The degree of embryo staining was observed under a stereomicroscope (SZ61; Olympus Corporation, Tokyo, Japan) to confirm seed viability. Both embryos and endosperms were recognized as viable only when stained red.

Seed size (20 seeds) and weight (100 seeds) were measured 4 times using Vernier calipers (Digimatic caliper; Mitutoyo, Kawasaki, Japan) and a microbalance (AS220, Radwag, Radon, Poland), respectively. The seed structures and cross-sections were observed using a stereomicroscope equipped with a CMOS camera (eXcope F630; Dixi Sci., Daejeon, Korea). The E:S ratio was calculated from the longitudinal section image of the seeds.

Initial germination tests were performed within 2 months of seed collection. Seeds were surface-sterilized with 1% sodium hypochlorite for 10 min and thoroughly washed with distilled water before sowing in a Petri dish on two layers of filter paper moistened with distilled water.

Seeds were incubated in an environment chamber set at 25/15°C, 16/8 h (light/dark), with a white LED (30 µmol·m^-2^s^-1^) light source. Seeds were also incubated in continuous darkness by wrapping Petri dishes with aluminum foil. The germination test lasted 30 days, and germination was recorded daily, except under dark conditions, which was not checked until the end of the experiment. All seed properties were investigated within 2 months after collection.

### Effect of seed coat scarification on germination

The effects of mechanical scarification on germination were determined simultaneously with the initial germination test. *A. elliptica* seeds were cut at the end of the 1-mm long seed coat using a razor blade, and non-scarified seeds were used as controls. After scarification, the seeds were sown on moist filter paper in Petri dishes and tested for germination under light/dark conditions, as described above.

Additionally, scarified seeds were sown in soil to determine whether they could produce normal seedlings. The seeds were sown in sterilized containers (sealed container, 318 × 230 × 175 mm, TAESUNG CNC Co., Ltd., Goyang, Korea) filled with a horticultural substrate (Hanareum; Shinsung Mineral Co., Ltd., Seongnam, Korea) and incubated under a light/dark cycle at 25/15°C. The containers were moistened with distilled water to prevent dehydration. Four replicates of 50 and 25 seeds were used for the Petri dishes and soil containers, respectively. The germination test in the Petri dishes lasted 30 days, and the germinated seeds were counted every day and removed from the dishes. The germination test in the soil was conducted during the same period, and seedling emergence and status were recorded. Germination was recorded when radicle protrusion was > 1 mm, and a normal seedling was defined as the normal emergence of both cotyledons and radicles.

### Dormancy break by stratification

We determined the effects of cold and warm plus cold stratification on dormancy break in *A. elliptica* seeds. Seeds were warm-stratified in the dark at 15/10°C (16/8 h, autumn) and then cold-stratified in the dark at 4°C (constant, winter). The conditions used in the experiment were set considering the ecological factors present in their natural habitats, such as seed maturation and germination. Seeds were first stratified for 4 or 8 weeks at 15/10°C (warm stratification, WS) and moved to a 4°C (cold stratification; CS) chamber for 4, 8, and 12 weeks. Other seed lots were subjected to CS at 4°C for 4, 8, and 12 weeks without WS. Mesh (filter) bags containing 50 seeds with 4 replications were placed in a sealed plastic box between layers of autoclaved wet pearlite (New Pershine No.2, GFC. Co., Ltd., Hongseong, Korea). Filter bags were removed from the box at 4-week intervals, and after seeds were surface-sterilized with 1% sodium hypochlorite for 10 min, they were sown in Petri dishes and tested for germination under a light/dark temperature of 25/15°C for 30 days.

### Effect of gibberellins on germination

We evaluated the effect of gibberellins on dormancy break in *A. elliptica* seeds. Various concentrations (100, 500, 1000, and 2,000 mg/L) of GA_3_ (Cas No. 77-06-5; Sigma-Aldrich) and GA_4+7_ (2:1, Cas No. 468-44-0/510-75-8; KisanBio, Seoul, Korea) were used to pre-soak *A. elliptica* seeds for 2 days at room temperature (25°C). Thereafter, the seeds were washed in tap water, sown in a Petri dish, and incubated under a light/dark cycle at 25/15°C for 30 days. Four replicates of 25 seeds were used for each concentration, and germination was monitored daily. Based on these results, the next experiment was conducted to determine the type and optimal concentration of GA for breaking dormancy.

In the second experiment, seeds were soaked in 500 mg/L GA_4+7_ and distilled water at room temperature (25°C) for 2, 4, 7, and 14 days. Five replicates of 20 seeds were used for each treatment, and the seeds were incubated under a light/dark cycle at 25/15°C for 30 days.

### Statistical analyses

SAS v9.4 (SAS Institute Inc., Cary, NC, USA) was used to calculate the mean and standard error for each treatment, and the analysis was performed using Duncan’s multiple range test at a significance level of *P* < 0.05.

## Results

### Seed properties

**Figure 1:**
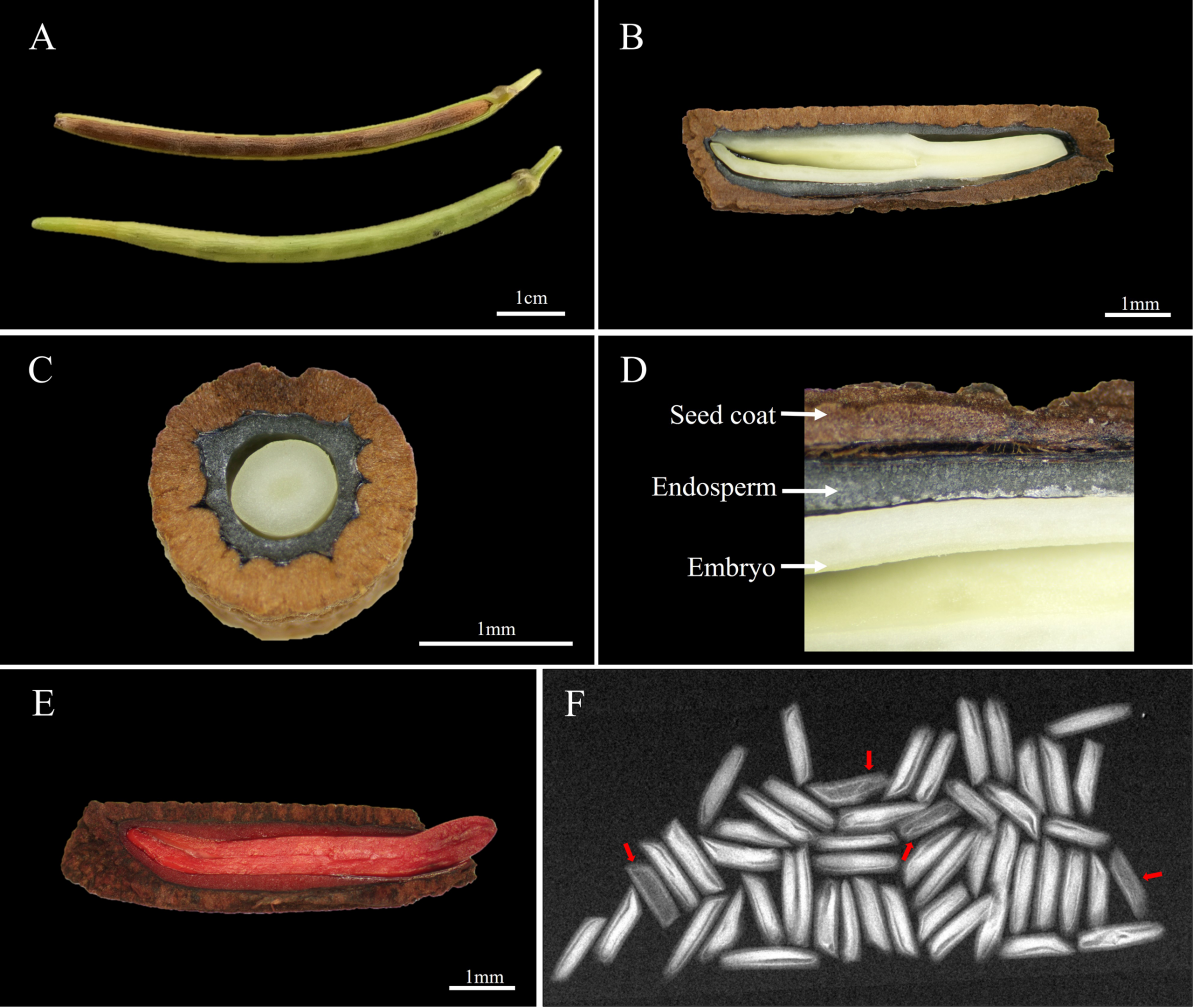
Morphological characteristics and viability of *Amsonia elliptica* seeds. **(A)**, arrangement of the seeds in the follicle; **(B)**, longitudinal section of seeds; **(C)**, horizontal section of seeds; **(D)**, the internal structure of seeds; **(E)**, scarified seed after soaking in tetrazolium solution; **(F)**, Fidelity of seeds confirmed by X-ray images. The red arrow represents empty seeds.

The *A. elliptica* seed is cylindrical in shape with a length and width of 9.1 ± 0.16 and 2.4 ± 0.05 mm, respectively, and the 100-seed weight was 1.6 ± 0.03 g (**Figures 1A, B**). Observation of the inner structure of seeds revealed a brown cork-textured seed coat and an embryo at the innermost layer, and an endosperm was identified between the coat and embryo (**Figures 1C, D**). The initial moisture content (MC) of seeds was 7.2 ± 0.65%, and during imbibition, the MC of both non-scarified and scarified seeds increased rapidly for 2 days, reaching 53.5 and 53.9%, respectively. However, no significant increase was observed after 2 days. The viability of seeds tested using tetrazolium solution was 90%, and X-ray analysis revealed that 94% of the seeds were filled, i.e., they had an embryo (**Figures 1E, F**).

### Effect of scarification on germination

**Figure 2:**
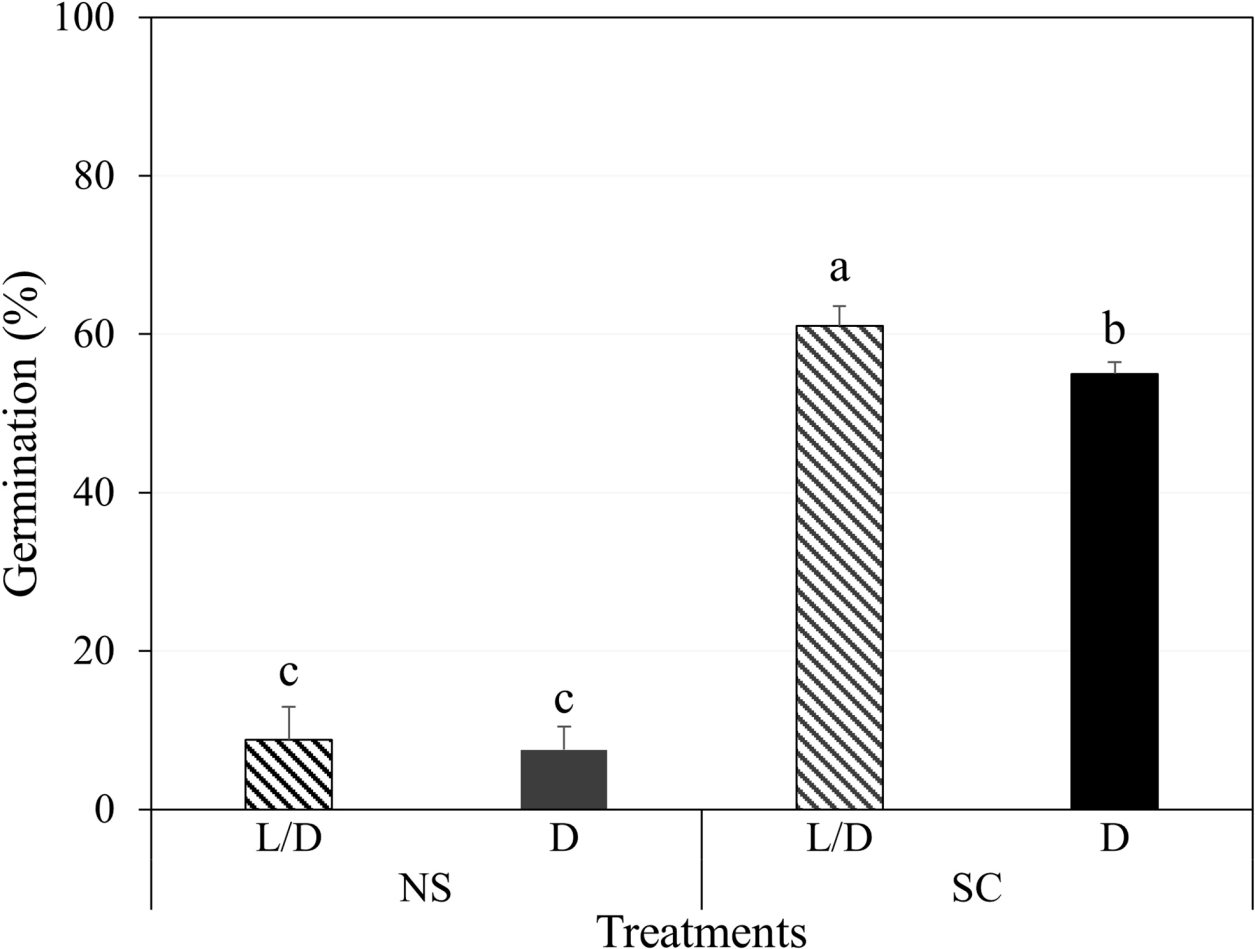
Initial germination of *Amsonia elliptica* seeds after 30 days. L/D, light/dark condition; D, dark condition; NS, non-scarified; SC, scarified. Bars represent standard error (*n* = 4). Different lowercase letters indicate a significant difference tested by Duncan’s multiple range test (P < 0.05).

**Figure 3:**
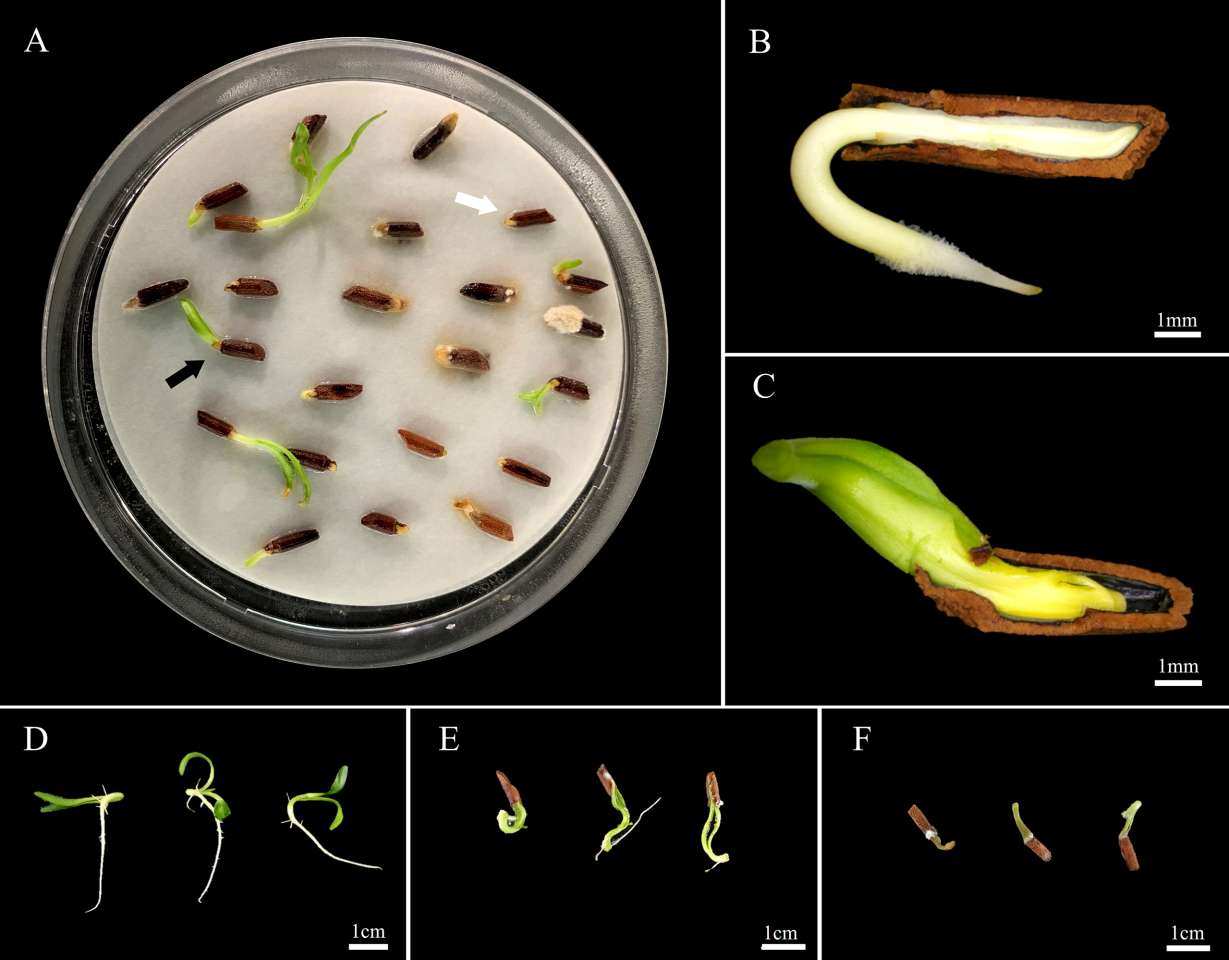
Normal and abnormal phenomena observed in germination and seedling emergence after *Amsonia elliptica* seed scarification. **(A)**, germination of scarified seeds observed in a Petri dish 14 days after sowing; **(B)**, the radicle protrudes first and can grow into normal seedlings; **(C)**, The cotyledon protrudes first, and the radicle fails to protrude, resulting in abnormal growth or death; **(D)**, normal seedlings; **(E)**, abnormal seedlings with arrested cotyledons; **(F)**, abnormal germination.

In Petri dishes, non-scarified seeds germinated to 8.8 and 7.5% under light/dark and dark conditions, respectively, while scarified seeds germinated to 61.0 and 55.0%, respectively (**Figure 2**). However, most of the germinated seeds either grew as abnormal seedlings or died (**Figure 3A**). Non-germinated seeds were tested for viability using 1% tetrazolium solution, and 90% of the seeds were viable.

In the soil, 31.7% of the scarified seeds produced emerged seedlings; however, 23.3% of the seedlings were abnormal (**Figure S1**). Scarified seeds exhibited a large proportion of abnormal phenomena in the germination process, such as seedlings with cotyledons caught by the seed coat and premature death after radicle protrusion (**Figures 3D–F**).

### Dormancy break according to stratification temperature and regime

**Figure 4:**
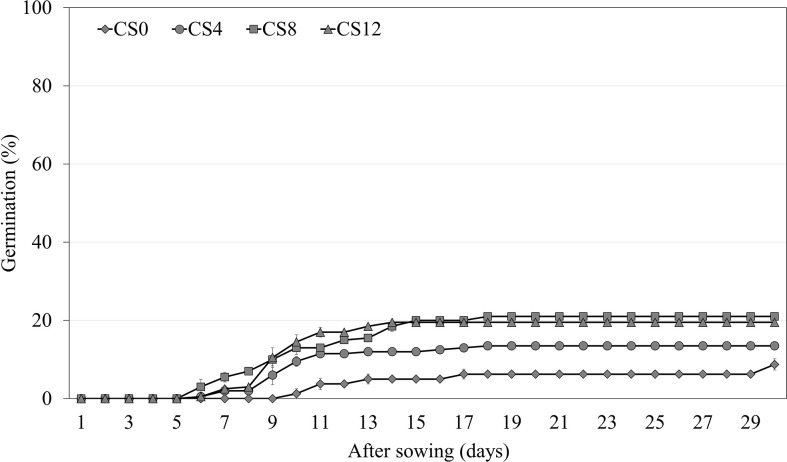
Germination of non-scarified *Amsonia elliptica* seeds in light/dark at 25/15 °C after cold stratification for 0–12 weeks. CS, cold stratification (4°C). Bars represent standard error (*n* = 4).

Cold stratification for 4 weeks significantly increased the germination of *A. elliptica* to 13.5% compared with the initial germination of 8.8% (**Figure 4**). As the length of the cold stratification treatment increased, germination increased to a maximum of only 21.0%; however, there was no significant difference between 8 and 12 weeks.

**Figure 5:**
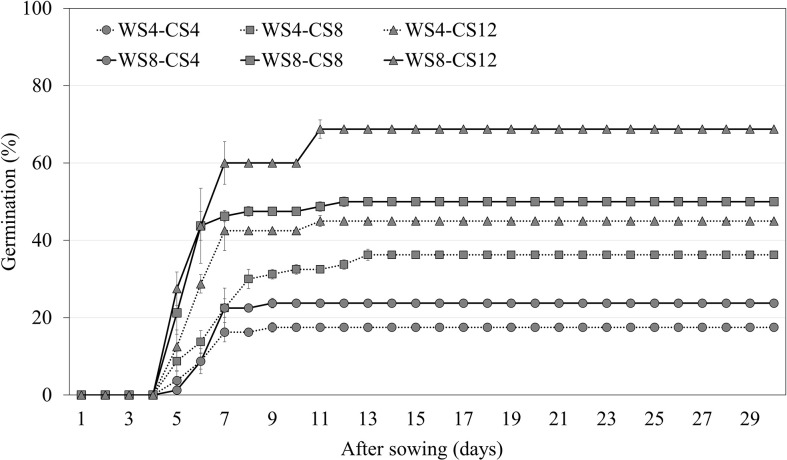
Germination of non-scarified *Amsonia elliptica* seeds at 25/15°C after combined stratification for indicated periods. WS, warm stratification (15/10°C, 16/8 h); CS, cold (4°C). Bars represent standard error (*n* = 4).

Warm scarification for 8 weeks, followed by 12 weeks of CS, increased the germination of *A. elliptica* seeds to 68.8% (**Figure 5**). Thus, WS followed by CS was more effective at breaking dormancy than CS alone. In addition, an increased WS period increased germination compared with cold stratification for the same period.

### Effect of gibberellins on germination

**Figure 6:**
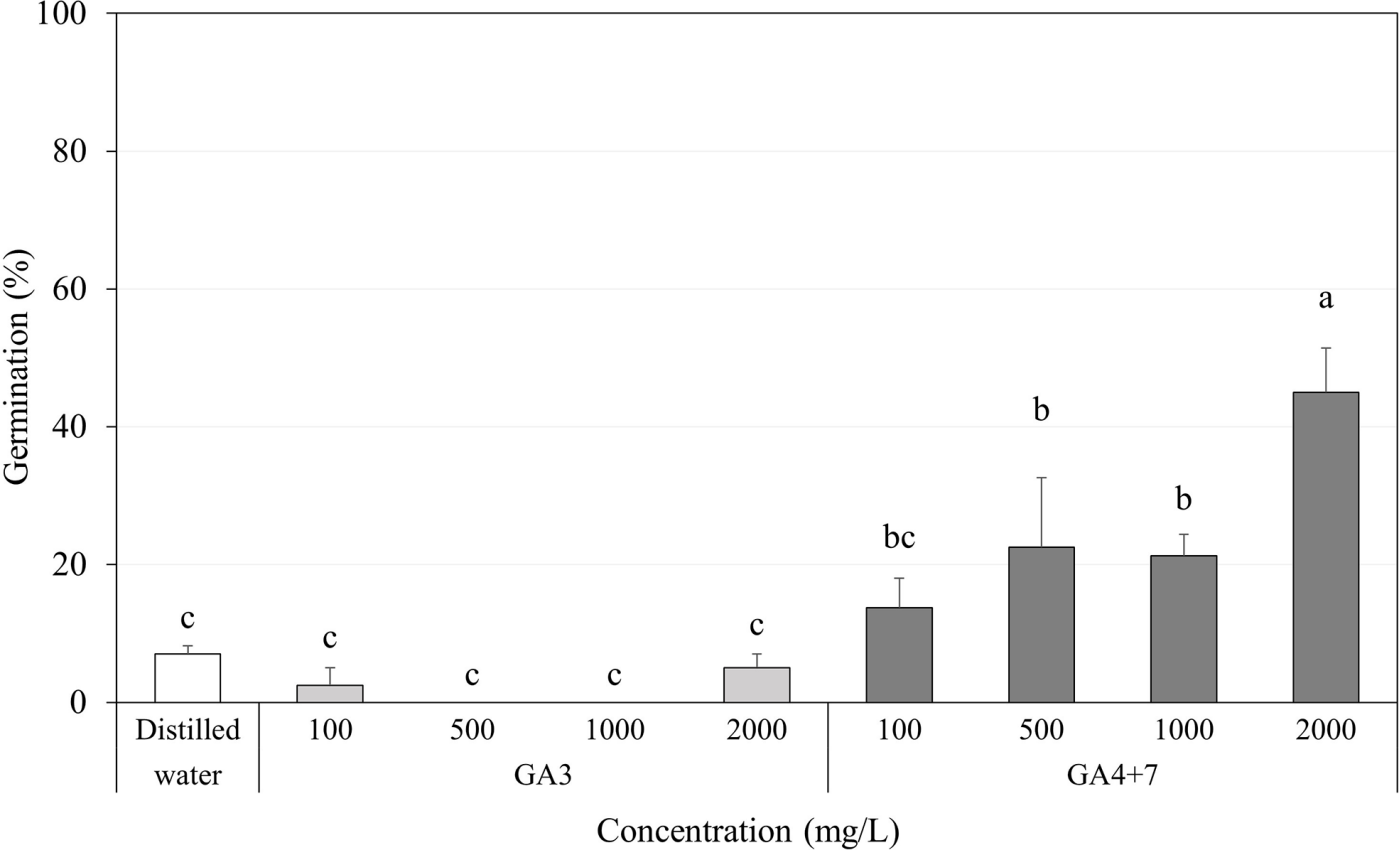
Percent germination of *Amsonia elliptica* seeds soaked in indicated concentrations of gibberellins (GAs) for 2 days. D.W., seeds soaked for 2 days in distilled water. Bars indicate standard errors (*n* = 4). Different lowercase letters indicate a significant difference tested by Duncan’s multiple range test (*P* < 0.05).

Regardless of the GA_3_ concentration in which the seeds were soaked, GA_3_ had little or no significant effect on increasing the germination percentage of *A. elliptica* seeds, exhibiting approximately the same effects as those observed in distilled water for 2 days (**Figure 6**).

In contrast, all concentrations of GA_4+7_ significantly promoted the germination of *A. elliptica* seeds, showing a gradual increase in germination with each increase in concentration. In particular, seeds soaked in 2,000 mg/L GA_4+7_ exhibited 45.0% germination.

### Effect of pre-soaking duration in GA_4+7_ on germination

**Figure 7:**
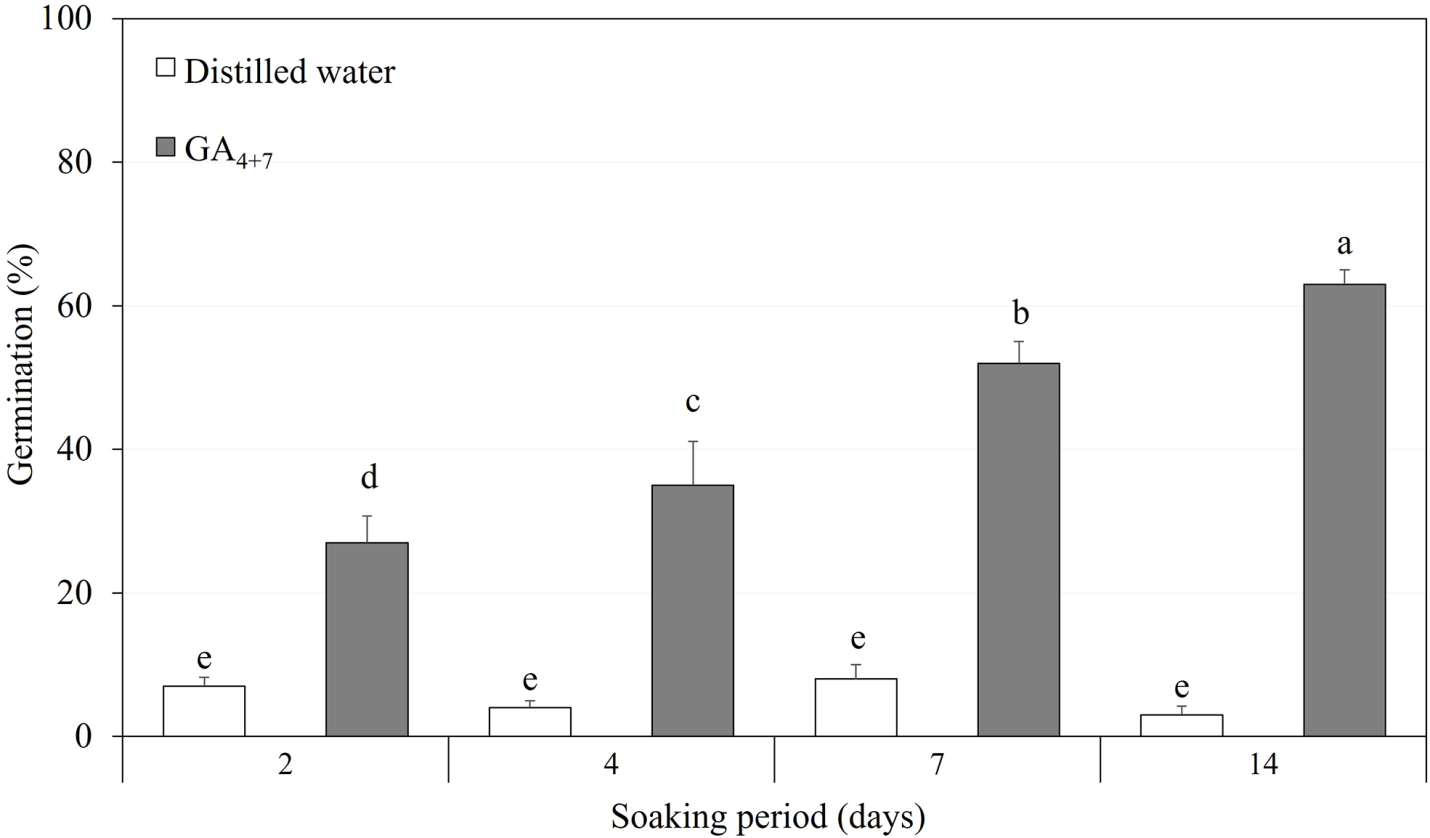
The comparison of overall percent germination of *Amsonia elliptica* seeds soaked either in distilled water or in 500 mg/L GA_4+7_ for 2 to 14 days. Bars indicate standard errors (*n* = 4). Different lowercase letters indicate a significant difference tested by Duncan’s multiple range test (*P* < 0.05).

After confirming the effect of GA_4+7_ on the germination of *A. elliptica* seeds, a moderate concentration of 500 mg/L was used to test the seeds, depending on the soaking period (**Figure 7**). Seeds soaked in 500 mg/L GA_4+7_ showed greatly improved germination compared with that of seeds soaked in water for the same period. In particular, seeds soaked for 14 days in GA_4+7_ exhibited 61.3% germination.

## Discussion

### *A. elliptica* has physiological dormancy

If seeds with a well-developed embryo and a water-permeable seed/fruit coat sown under suitable germination conditions fail to germinate within approximately 4 weeks, they are either nonviable or have physiological dormancy. *A. elliptica* seeds, whose viability was confirmed, did not germinate within 4 weeks in the initial germination test but produced fully developed embryos (**Figure 1B**). Thus, seeds of this species have PD, which is a physiological-inhibiting mechanism that prevents germination due to insufficient embryonic growth potential for germination. Therefore, scarification may promote germination by releasing mechanical restrictions on embryos. Patents related to the promotion of *A. elliptica* seed germination indicate that the seeds cannot absorb water, and germination is promoted by treatment with sodium hypochlorite or sulfuric acid to chemically scarify the seed coat (Choi and Jung, 2017). However, in the imbibition test performed in our study, the moisture content of non-scarified *A. elliptica* seeds increased to 53.5% after immersion in water. Thus, the seeds were water-permeable and did not exhibit physical dormancy.

Germination of the water-permeable seeds of *A. tabernaemontana* (Martin et al., 2013) and other species, such as *Cucumis melo* (Welbaum et al., 1990), *Fraxinus excelsior* (Finch-Savage and Clay, 1997), and *Lycopersicon esculentum* (Downie et al., 2003) is increased by scarification of the seed coat, which removes the mechanical restriction on the embryo. Nikolaeva (1977) classified dormancy induced by mechanical restriction due to the seed-covering layer as mechanical dormancy. However, according to Baskin and Baskin (2004), suppression of germination by mechanical resistance is a physiologically dormant state in which seeds cannot germinate due to low growth potential (‘expansive force’) rather than dormancy induced by the covering layer. After the PD is broken, the embryo has sufficient growth potential to overcome the mechanical resistance of the covering layer. However, in the initial germination test, scarified seeds of *A. elliptica* sown in Petri dishes did not germinate normally, despite the removal of the mechanical resistance of the covering layer (**Figure 3A**). In normal germination, the radicle usually protrudes first (**Figure 3B**); however, in scarified seeds, the cotyledons protrude first, and the radicle does not protrude (**Figure 3C**) or the cotyledons are arrested by the seed coat and grow into abnormal seedlings (**Figures 3D–F**). For the scarified seeds sown in the soil, although radicles protruded from the seed coat, only a few seeds grew into normal seedlings (**Figure S1**). This phenomenon was assumed to be caused by the insufficient growth potential of the embryo. Therefore, it was confirmed that artificial scarification could harm both seeds and seedlings. This results in abnormal germination following scarification, indirectly confirming that the cause of germination inhibition is not in the coat but in the seed (embryo).

### Alleviated physiological dormancy by stratification

The most common dormancy in terrestrial seed plants is PD (Baskin and Baskin, 2014), which is usually alleviated by temperature (cold moist stratification, warm moist stratification, after-ripening), hormones, or chemical treatments (Baskin and Baskin, 2004; Baskin and Baskin, 2014; Cho et al., 2020; Shao et al., 2021; Baskin and Baskin, 2022). Physiological dormancy is divided into two subclasses: regular and epicotyl. In regular PD, the shoot emerges within a few days of radicle emergence, but in epicotyl PD there is a delay of several months between radicle and shoot emergence (Baskin and Baskin, 2022). As confirmed by the initial germination test, *A. elliptica* seeds have regular PD, subdivided into three levels: non-deep, intermediate, and deep. Dormancy break in seeds with non-deep PD usually occurs during 8–10 weeks of warm or cold stratification, depending on the species. However, cold stratification of *A. elliptica* seeds resulted in low germination percentages, suggesting that dormancy was either intermediate or deep.

If seeds of temperate-zone species have intermediate PD, warm stratification followed by cold stratification is effective in breaking dormancy, and seeds usually respond to treatment with GA_3_. Without warm stratification pretreatment, seeds require 12–16 weeks of cold stratification to germinate. If seeds of temperate-zone species have deep PD, they require 12–24 weeks of cold stratification for dormancy break, and they do not respond to treatment with GA_3_ (Baskin and Baskin, 2022). For *A. elliptica* seeds, warm stratification followed by cold stratification dramatically increased the germination percentage (**Figure 5**). The improved germination of seeds receiving warm + cold stratification compared with that of seeds undergoing only cold stratification suggests that the seeds have an intermediate PD. In the natural habitat, *A. elliptica* seeds are exposed to warm stratification for at least 4 weeks in autumn before they are exposed to cold stratification in winter.

### Gibberellins rapidly break seed dormancy without stratification

Exogenous hormones improve seed germination in many species in past times (Stowe and Yamaki, 1957). In particular, gibberellins are commonly used to improve the germination, growth and flowering of seeds and plants, usually in concert with abscisic acid and auxins, by up- or downregulating genes related to gibberellin biosynthesis (Wolbang et al., 2004; Frigerio et al., 2006; Seo et al., 2006; O’Neill et al., 2010; Yaish et al., 2010). There are many gibberellin structures, and their biological activity differs depending on their form (Mander, 1992; Shu et al., 2016). Among them, GA_3_ is best known in seed germination and dormancy, and it is also a criterion for classifying PD subdivision according to its response (Baskin and Baskin, 2014). If *A. elliptica* seeds exhibit an intermediate PD, GA_3_ is expected to promote germination. However, we found that GA_3_ did not promote germination, while combining GA_4_ and GA_7_ was effective in breaking dormancy and did so in the absence of cold stratification (**Figure 6**). GA_4_ and GA_7_ are synthesized from GA_9_ reaction with GA3ox, and GA_4_ and GA_7_ are the bioactive forms of gibberellin. However, they are not as well known as GA_3_ in dormancy and germination. GA_4_ has been identified as the major form of gibberellin in *Arabidopsis*, Cucurbitaceae, and rice (John et al., 2016). Increased GA_4_ positively regulates the seed germination of *Brassica parachinensis* (Chen et al., 2021), *Glycine max* (Chen et al., 2020), and *Syngonanthus verticillatus* (Barreto et al., 2020), while reducing GA_4_ can inhibit germination. The reason *A. elliptica* seeds do not react to GA_3_ could be that the 13-hydroxylation pathway negatively affects its biological activity (Magome et al., 2013). Therefore, the percentage germination was even lower than that of the control seeds. Another hypothesis is that GA_4_ or GA_7_ contribute to endosperm weakening, which may restrain embryo expansion in *A. elliptica* seeds (Brown and Bridglall, 1987). Bioactive gibberellins are major contributors to endosperm weakening in *Lepidium sativum* seeds (Oracz et al., 2012; Voegele et al., 2012). In a study by Oracz et al. (2012), GA_4_ was also involved in weakening the structure enveloping the embryo. Therefore, inhibition of *A. elliptica* seed germination is assumed to be eliminated by serial or spontaneous gibberellin actions. However, our study did not investigate the mechanisms, by which gibberellins promote germination by breaking dormancy.

## Conclusions

*Amsonia elliptica* seeds have intermediate PD that is broken by warm conditions followed by cold stratification or by treatment with a mixture of GA_4_ and GA_7_. Although the seeds are water permeable, scarification of the seed coat promotes germination by eliminating the mechanical resistance of the embryo. However, a high percentage of the resulting seedlings are abnormal. This phenomenon means that PD in *A. elliptica* is not simply due to mechanical resistance but is caused by insufficient embryonic growth potential. After dormancy was broken by warm plus cold stratification or by treatment with a combination of GA_4_ and GA_7_, the growth of seedlings was normal, indicating that dormancy breaking in *A. elliptica* seeds increases the growth potential, so that embryos can overcome their mechanical resistance on their own. Dormancy break following warm plus cold stratification in the laboratory simulates the treatment seeds receive in their native habitat due to seasonal changes in temperature between the time of dispersal in autumn and germination in spring. However, this is a long period of time, and we found that normal seedlings can be produced over a relatively short period by soaking the seeds in 500 mg/L GA_4+7_ for 2 to 14 days and then incubating them under simulated spring temperatures (25/15°C). Thus, treatment with GA_4+7_ not only breaks intermediate PD but does so within approximately 1 month from the start of seed soaking in GA_4+7_. Treatment with GA_4+7_ effectively shortened the period required for dormancy break, enabling rapid germination and seedling production. Our methods can be used to produce *A. elliptica* for restoration, and the wide use of this species can be expected in various fields, such as medicine and the horticultural industry, through mass propagation.

## Data availability statement

The original contributions presented in the study are included in the article/**Supplementary Material**. Further inquiries can be directed to the corresponding author.

## Author contributions

SL, KP, and J-SC: conceptualization. B-KJ, and BJ: data curation. SL, HL: methodology. SL, KP: writing–original draft preparation. CB and J-SC: writing–review and editing. All authors contributed to the article and approved the submitted version.

## Funding

This work was supported by the National Research Foundation of Korea (NRF) grant funded by the Korea government (MSIT) (No. 2021R1G1A1007156).

## Acknowledgments

We thank the National Research Foundation of Korea for funding support.

## Conflict of interest

Author SL is employed by The Kiban co. Ltd.,

The remaining authors declare that the research was conducted in the absence of any commercial or financial relationships that could be construed as a potential conflict of interest.

## Publisher’s note

All claims expressed in this article are solely those of the authors and do not necessarily represent those of their affiliated organizations, or those of the publisher, the editors and the reviewers. Any product that may be evaluated in this article, or claim that may be made by its manufacturer, is not guaranteed or endorsed by the publisher.

## Supplementary material

Supplementary Figure 1 Normal seedlings and abnormal germination of scarified seeds of *Amsonia elliptica* germinated in Petri dishes **(A)** and soil **(B)**. The black arrow is a normal seedling.

## References

[B1] Barreto L. C. Herken D. Silva B. M. Munné-Bosch S. Garcia Q. S. (2020). ABA and GA_4_ dynamic modulates secondary dormancy and germination in *Syngonanthus verticillatus* seeds. Planta 251 (4), 1–10. doi: 10.1007/s00425-020-03378-2 32221719

[B2] Baskin J. M. Baskin C. C. (2004). A classification system for seed dormancy. Seed. Sci. Res. 14, 1–16. doi: 10.1079/SSR2003150

[B3] Baskin C. Baskin J. M. (2014). Seeds: Ecology, biogeography, and evolution of dormancy and germination (San Diego: Academic Press).

[B4] Baskin J. M. Baskin C. C. (2021). The great diversity in kinds of seed dormancy: a revision of the nikolaeva–baskin classification system for primary seed dormancy. Seed. Sci. Res. 31, 249–277. doi: 10.1017/S096025852100026X

[B5] Baskin C. C. Baskin J. M. (2022). Invited review: mimicking the natural thermal environments experienced by seeds to break physiological dormancy to enhance seed testing and seedling production. Seed. Sci. Technol. 50, 1–2. doi: 10.15258/sst.2022.50.1.s.02

[B6] Brown N. A. C. Bridglall S. S. (1987). Preliminary studies of seed dormancy in *Datura stramonium* . S. Afr. J. Bot. 53, 107–109. doi: 10.1016/S0254-6299(16)32626-6

[B7] Chen J. Z. Huang X. L. Xiao X. F. Liu J. M. Liao X. F. Sun Q. W.. (2022). Seed dormancy release and germination requirements of *Cinnamomum migao*, an endangered and rare woody plant in southwest China. Front. Plant Sci. 13, 770940–770940. doi: 10.3389/fpls.2022.770940 35154219 PMC8828499

[B8] Chen B. X. Peng Y. X. Yang X. Q. Liu J. (2021). Delayed germination of *Brassica parachinensis* seeds by coumarin involves decreased GA_4_ production and a consequent reduction of ROS accumulation. Seed. Sci. Res. 31 (3), 224–235. doi: 10.1017/S0960258521000167

[B9] Chen F. Zhou W. Yin H. Luo X. Chen W. Liu X.. (2020). Shading of the mother plant during seed development promotes subsequent seed germination in soybean. J. Exp. Bot. 71 (6), 2072–2084. doi: 10.1093/jxb/erz553 31925954 PMC7242070

[B10] Choi K. E. Jung M. J. (2017). “Germination method of bluestar seed,” in Korea Patent no. 10-2017-0078854 (Daejeon: Korean Intellectual Property Office). doi: 10.8080/1020170078854

[B11] Cho J. S. Jang B. K. Lee S. M. Lee I. J. Lee C. H. (2020). Factors affecting the dormancy and germination of bleeding heart [*Lamprocapnos spectabilis* (L.) fukuhara] seeds. Plant Biol. (Stuttg). 22, 514–521. doi: 10.1111/plb.13089 31965672

[B12] Cochrane J. A. Crawford A. D. Monks L. T. (2007). The significance of ex situ seed conservation to reintroduction of threatened plants. Aust. J. Bot. 55, 356–361. doi: 10.1071/BT06173

[B13] Downie B. Gurusinghe S. Dahal P. Thacker R. R. Snyder J. C. Nonogaki H.. (2003). Expression of a GALACTINOL synthase gene in tomato seeds is up-regulated before maturation desiccation and again after imbibition whenever radicle protrusion is prevented. Plant Physiol. 131, 1347–1359. doi: 10.1104/pp.016386 12644684 PMC166894

[B14] Finch-Savage W. E. Clay H. A. (1997). The influence of embryo restraint during dormancy loss and germination of fraxinus excelsior seeds, in basic and applied aspects of seed biology. Eds. Ellis R. H. Black M. Murdoch A. J. Hong T. D. (Dordrecht: Springer), 245–253.

[B15] Frigerio M. Alabadí D. Pérez-Gómez J. García-Cárcel L. Phillips A. L. Hedden P.. (2006). Transcriptional regulation of gibberellin metabolism genes by auxin signaling in *Arabidopsis* . Plant Physiol. 142, 553–563. doi: 10.1104/pp.106.084871 16905669 PMC1586059

[B16] Hassani S. B. Saboora A. Radjabian T. Fallah Husseini H. (2009). Effects of temperature, GA_3_ and cytokinins on breaking seed dormancy of *Ferula assa-foetida* L. Iranian. J. Sci. Technol. (Sciences). 33 (1), 75–85.

[B17] Hawkins B. Sharrock S. Havens K. (2008). Plants and climate change: Which future? (Richmond: Botanic Gardens Conservation International).

[B18] Hay F. R. Probert R. J. (2013). Advances in seed conservation of wild plant species: a review of recent research. Conserv. Physiol. 1, cot030. doi: 10.1093/conphys/cot030 27293614 PMC4806614

[B19] He X. Q. Wang Y. R. Hu X. W. Baskin C. C. Baskin J. M. Lv Y. Y. (2016). Seed dormancy and dormancy-breaking methods in *Leymus chinensis* (Trin.) Tzvel.(Poaceae). Grass. Forage. Sci. 71 (4), 641–648. doi: 10.1111/gfs.12220

[B20] Hilhorst H. W. M. (1995). A critical update on seed dormancy. i. primary dormancy1. Seed. Sci. Res. 5, 61–73. doi: 10.1017/S0960258500002634

[B21] Jang B. K. Park K. Lee S. Y. Lee H. Song S. K. Kim J.. (2023). Comparison of the seed dormancy and germination characteristics of six *Clematis* species from south Korea. Sci. Hortic 307, 111488. doi: 10.1016/j.scienta.2022.111488

[B22] John J. R. Asemeh M. Amelia H. B. Laura J. Q. Erin L. M. (2016). “Interactions between gibberellins and other hormones,” in Annual plant reviews, the gibberellins, vol. Vol. 49) . Eds. Hedden P. Thomas S. G. (NJ: John Wiley & Sons), 229–252.

[B23] Kim Y. J. Kim B. H. Lee S. Y. Kim M. S. Park C. S. Rhee M. S.. (2006). Screening of medicinal plants for development of functional food ingredients with antiobesity. Appl. Biol. Chem. 49, 221–226.

[B24] Kramer A. T. Havens K. (2009). Plant conservation genetics in a changing world. Trends Plant Sci. 14, 599–607. doi: 10.1016/j.tplants.2009.08.005 19748300

[B25] Lee S. Y. Rhie Y. H. Kim K. S. (2018). Dormancy breaking and germination requirements of seeds of *Thalictrum uchiyamae* (Ranunculaceae) with underdeveloped embryos. Sci. Hortic. 231, 82–88. doi: 10.1016/j.scienta.2017.12.004

[B26] Li D. Z. Pritchard H. W. (2009). The science and economics of ex situ plant conservation. Trends Plant Sci. 14, 614–621. doi: 10.1016/j.tplants.2009.09.005 19818672

[B27] Magome H. Nomura T. Hanada A. Takeda-Kamiya N. Ohnishi T. Shinma Y.. (2013). CYP714B1 and CYP714B2 encode gibberellin 13-oxidases that reduce gibberellin activity in rice. Proc. Natl. Acad. Sci. U. S. A. 110, 1947–1952. doi: 10.1073/pnas.1215788110 23319637 PMC3562828

[B28] Mander L. N. (1992). The chemistry of gibberellins: an overview. Chem. Rev. 92 (4), 573–612. doi: 10.1021/cr00012a005

[B29] Martin M. T. Chappell M. R. Opie L. R. (2013). Scarification and germination of *Amsonia tabernaemontana* (Walt.). Seeds. J. Environ. Hortic. 31, 54–58. doi: 10.24266/0738-2898.31.1.54

[B30] Nikolaeva M. G. (1977). “Factors controlling the seed dormancy pattern”. In The physiology and biochemistry of seed dormancy and germination. Khan A. A, ed. (North-Holland, Amsterdam:North Holland Biomedical Press), 51–74.

[B31] Nikolaeva M. G. (2004). On criteria to use in studies of seed evolution. Seed. Sci. Res. 14, 315–320. doi: 10.1079/SSR2004185

[B32] O’Neill D. P. Davidson S. E. Clarke V. C. Yamauchi Y. Yamaguchi S. Kamiya Y.. (2010). Regulation of the gibberellin pathway by auxin and DELLA proteins. Planta 232, 1141–1149. doi: 10.1007/s00425-010-1248-0 20706734

[B33] Oracz K. Voegele A. Tarkowská D. Jacquemoud D. Turecková V. Urbanová T.. (2012). Myrigalone a inhibits *Lepidium sativum* seed germination by interference with gibberellin metabolism and apoplastic superoxide production required for embryo extension growth and endosperm rupture. Plant Cell Physiol. 53, 81–95. doi: 10.1093/pcp/pcr124 21908442

[B34] Sauerwein M. Ishimaru K. Shimomura K. (1991). Indole alkaloids in hairy roots of *Amsonia elliptica* . Phytochemistry 30, 1153–1155. doi: 10.1016/S0031-9422(00)95193-8

[B35] Seo M. Hanada A. Kuwahara A. Endo A. Okamoto M. Yamauchi Y.. (2006). Regulation of hormone metabolism in *Arabidopsis* seeds: phytochrome regulation of abscisic acid metabolism and abscisic acid regulation of gibberellin metabolism. Plant J. 48, 354–366. doi: 10.1111/j.1365-313X.2006.02881.x 17010113

[B36] Shao C. Wang G. Ding X. Yang C. Yan M. (2021). Physiological and biochemical characteristics of cold stratification to overcome morphophysiological dormancy in *Glehnia littoralis* seed. Seed. Sci. Technol. 49, 19–24. doi: 10.15258/sst.2021.49.1.03

[B37] Shu K. Liu X. D. Xie Q. He Z. H. (2016). Two faces of one seed: hormonal regulation of dormancy and germination. Mol. Plant 9 (1), 34–45. doi: 10.1016/j.molp.2015.08.010 26343970

[B38] Stowe B. B. Yamaki T. (1957). The history and physiological action of the gibberellins. Annu. Rev. Plant Physiol. 8, 181–216. doi: 10.1146/annurev.pp.08.060157.001145

[B39] Urbanova T. Leubner-Metzger G. (2016). “Gibberellins and seed germination,” in Annual plant reviews, volume 49: The gibberellins eds,Hedden P. Thomas S. G (Hoboken, New Jersey: Wiley), 253–284.

[B40] Vishal B. Kumar P. P. (2018). Regulation of seed germination and abiotic stresses by gibberellins and abscisic acid. Front. Plant Sci. 9, 838. doi: 10.3389/fpls.2018.00838 29973944 PMC6019495

[B41] Voegele A. Graeber K. Oracz K. Tarkowská D. Jacquemoud D. Turečková V.. (2012). Embryo growth, testa permeability, and endosperm weakening are major targets for the environmentally regulated inhibition of *Lepidium sativum* seed germination by myrigalone a. J. Exp. Bot. 63, 5337–5350. doi: 10.1093/jxb/ers197 22821938 PMC3431005

[B42] Walther G. R. Post E. Convey P. Menzel A. Parmesan C. Beebee T. J.. (2002). Ecological responses to recent climate change. Nature 416, 389–395. doi: 10.1038/416389a 11919621

[B43] Welbaum G. E. Tissaoui T. Bradford K. J. (1990). Water relations of seed development and germination in muskmelon (*Cucumis melo* l.): III. sensitivity of germination to water potential and abscisic acid during development. Plant Physiol. 92, 1029–1037. doi: 10.1104/pp.92.4.1029 16667367 PMC1062412

[B44] Wolbang C. M. Chandler P. M. Smith J. J. Ross J. J. (2004). Auxin from the developing inflorescence is required for the biosynthesis of active gibberellins in barley stems. Plant Physiol. 134, 769–776. doi: 10.1104/pp.103.030460 14730077 PMC344552

[B45] Yaish M. W. El-Kereamy A. Zhu T. Beatty P. H. Good A. G. Bi Y. M.. (2010). The APETALA-2-Like transcription factor OsAP2-39 controls key interactions between abscisic acid and gibberellin in rice. PloS Genet. 6, e1001098. doi: 10.1371/journal.pgen.1001098 20838584 PMC2936520

[B46] Yang J. C. Park S. H. Ha S. G. Lee Y. M. (2012). The flora of vascular plants in daecheong island, south Korea. Korean. J. Plant Resour. 25, 31–47. doi: 10.7732/kjpr.2012.25.1.031

