# Supplementary figures and images for: Exogenous gibberellin can effectively and rapidly break intermediate physiological dormancy of Amsonia elliptica seeds
Source: Front Plant Sci. 2022 Oct 26;13:1043897. doi: 10.3389/fpls.2022.1043897 (PMC9643720; doi:10.3389/fpls.2022.1043897)

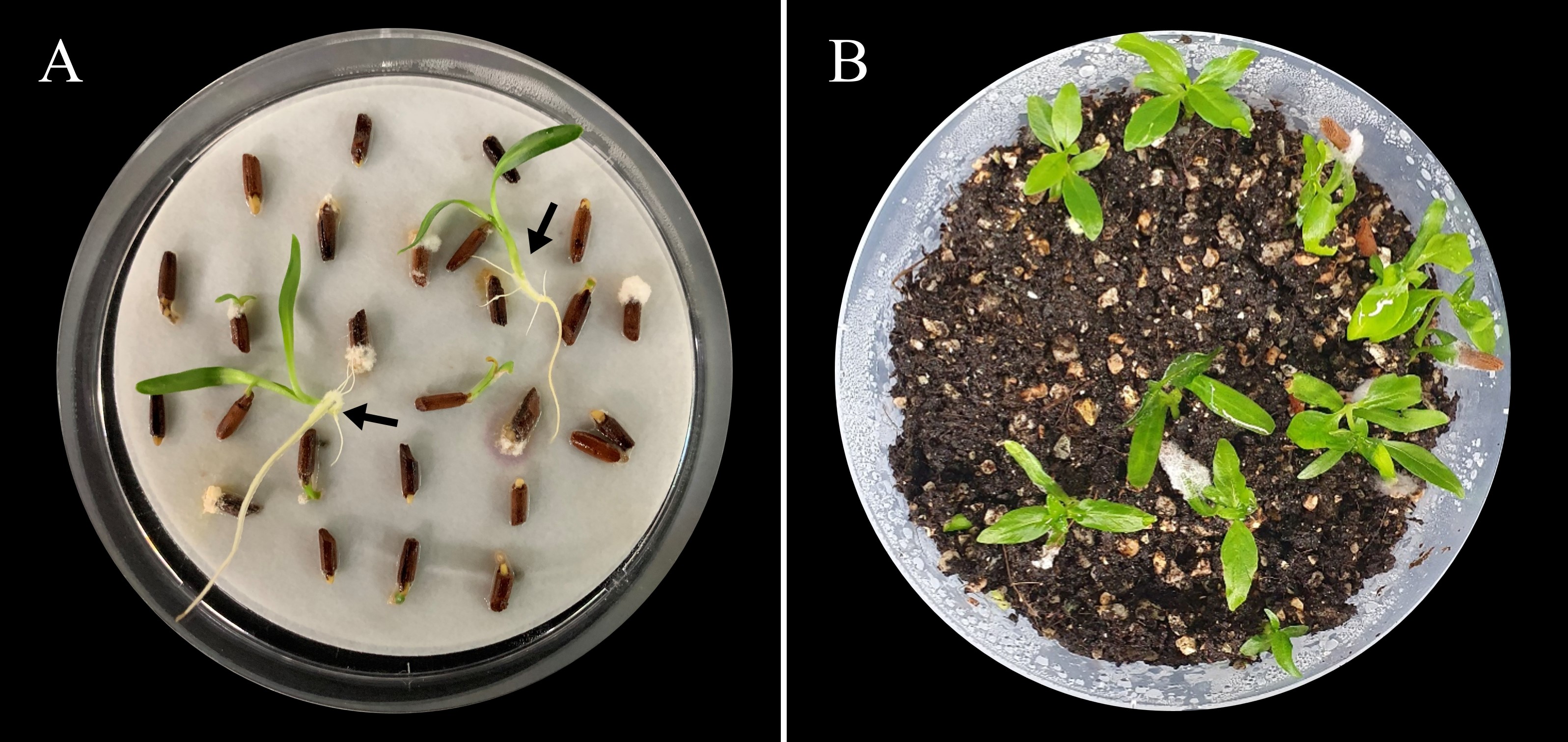

Supplement: Supplementary Figure 1 — Normal seedlings and abnormal germination of scarified seeds of Amsonia elliptica germinated in Petri dishes (A) and soil (B). The black arrow is a normal seedling. [file Image_1.jpeg]
